# Nutritional Composition of *Bombyx mori* Pupae: A Systematic Review

**DOI:** 10.3390/insects13070644

**Published:** 2022-07-17

**Authors:** Luca Tassoni, Silvia Cappellozza, Antonella Dalle Zotte, Simone Belluco, Pietro Antonelli, Filippo Marzoli, Alessio Saviane

**Affiliations:** 1 Consiglio per la Ricerca in Agricoltura e l’Analisi dell’Economia Agraria, Centro di Ricerca Agricoltura e Ambiente (CREA-AA), 35143 Padova, Italy; silvia.cappellozza@crea.gov.it (S.C.); alessio.saviane@crea.gov.it (A.S.); 2 Department of Animal Medicine, Production and Health, University of Padova, Agripolis, Viale dell’Università 16, 35020 Padova, Italy; antonella.dallezotte@unipd.it; 3 IZSVe, Istituto Zooprofilattico Sperimentale delle Venezie, Viale dell’Università 10, 35020 Padova, Italy; sbelluco@izsvenezie.it (S.B.); pantonelli@izsvenezie.it (P.A.); fmarzoli@izsvenezie.it (F.M.)

**Keywords:** *Bombyx mori*, mulberry silkworm pupae, nutritional composition

## Abstract

**Simple Summary:**

The mulberry silkworm (*Bombyx mori*) is a domesticated insect traditionally reared to produce silk. Its pupae are historically eaten in Asian countries and are obtained as waste products from the silk reeling industry. Pupae are a promising novel food in Western countries as well as a source of proteins, lipids, and minerals. Several varied results are reported in the literature regarding the nutrient composition of silkworm pupa, and several factors must be considered when comparing the research. Some of the variables that could affect the pupal nutritional content include rearing techniques, diets, silkworm strains, killing, and drying techniques. This literature systematic review identifies the most important research areas and aids authorities and producers in the evaluation and development of silkworm pupae for novel uses.

**Abstract:**

As insects have started to enter the eating habits of Western countries, an increasing amount of literature regarding the mulberry silkworm (*Bombyx mori*) prospective application as food has been published. Despite this growing interest, there is currently no systematic review of silkworm nutritional composition available. In this paper, we performed a systematic review of the recent available literature on the nutrient composition of mulberry silkworm pupae. After screening the titles and abstracts of 14,008 studies retrieved from three scientific databases, data about nutrients was extracted from 29 selected papers, together with their related variables. This systematic review provides an overview of the variety of data reported in the literature and highlights that many elements contribute to hindering a sound comparison of the different nutritional values reported for silkworm pupae. The observed variability of the composition data reported could be due to differences in diet, strains, pretreatments, and origin of the silkworm analyzed. However, all these variables were not always available and should be reported in future studies to simplify the data comparison.

## 1. Introduction

The only fully domesticated insect among those reared by humans is the mulberry silkworm (*Bombyx mori* L., 1758), which was first raised in captivity around 7500 years ago [1]. Its ability to generate silk is what gives it its significance, although it also has applications as food or feed, a pet, and a model organism in scientific research [2]. Furthermore, the silkworm has been used as a functional bioreactor to create a variety of molecules, mostly those with pharmaceutical implications [3].

Pupae are the most commonly employed life stage for food or feed since they make up around 60% of the dry cocoon weight [4] and are the principal by-product of the silk industry. They can be used as food or feed, which helps to support circular production chains and prevent the release of potentially harmful waste into the environment. The consumption of silkworm pupae is already widespread in Asia, particularly in India, China, Japan, South Korea, and Thailand [5,6,7,8,9], where they are valued as street food, or as a nutraceutical in traditional medicine [10,11,12].

Before consumption, it is important to weigh the dangers associated with allergens, microorganisms, or chemicals, in addition to the nutritional advantages of silkworm pupae. Regarding Europe, an EFSA (the European Food Safety Agency) opinion [13] explored the possibility of silkworms as a novel food, reflecting the growing interest in this topic in Western nations.

In this systematic review, we concentrated on analyzing and summarizing the literature on the nutritional composition of *B. mori* pupae (further called SP for “silkworm pupae”). There are reviews addressing silkworm composition [14], but a systematic review addressing silkworm pupa composition is lacking. The main distinction between this systematic review and other reviews is that it compares all the data that are currently available about SP published in the searched literature in the declared time interval, allowing us to understand the potential and restrictions of SP as a food. On the other hand, we may propose some hypotheses regarding the main aspects that may affect the nutritional value of SP based on a thorough comparison of the findings of various authors.

Authorities may find this systematic review useful when they evaluate SP and its derivative products as novel foods. Understanding the potential technology uses and the impact of the technological replacement of conventional nutrient sources in novel formulations could potentially be helpful for food producers. Additionally, this analysis could serve as a basis for future research looking into novel approaches to manipulate SP composition.

## 2. Materials and Methods

This systematic review seeks to provide a food-based characterization of the nutritional composition of mulberry SP (*B. mori*). All articles published in peer-reviewed journals in the languages of English, French, and Spanish were taken into consideration for this scope at the onset of our study without regard to publication dates.

Using the keywords listed in Table 1, we conducted searches in PUBMED, Web of Science Core Collection, and EMBASE (Title/Abstract, Topic (TS), and Title, Abstract, Author keywords, respectively). The keywords also contained terms pertaining to the allergic and security aspects of SP. The records pertaining to these subjects were located and used for a second systematic review with a focus on these specific concerns. The search was performed on 14 May 2020, and it was updated on 5 May 2021, with a time limit beginning on 1 January 2020, to include all the documents published in the meantime. We utilized the software EPPI-4 Reviewer [15] to examine the retrieved records

There were two main phases to the selection process. Six researchers (S.B., F.M., A.P., L.T., A.S., and S.C.) conducted the first phase using a double-screening approach, in which two researchers separately classified each record to determine whether it related to the study topic “composition” or “safety” and to determine whether it was a review.

This step’s inclusion criteria were as follows: (1) the language used had to be either English, French, or Spanish; (2) the data had to be taken directly from research publications and not from other reviews. All the records with unclear titles or abstracts were included for evaluation in the following stage. Based on the titles and abstracts, the selected studies were then split into two groups: those dealing with nutritional composition and those dealing with biological/chemical risk related to SP use. Three reviewers (S.B., F.M., and P.A.) did this classification.

The second phase concentrated on original papers addressing SP composition. The task involved classifying and evaluating each paper’s data typology in full text for each record. Then, pertinent information was retrieved and listed in tables. We specifically found publications that discussed the following topics: (1) the overall chemical composition (macronutrients and ash), (2) the AA profile, (3) the FA profile, and (4) the composition of the macro and microelements.

The following additional exclusion criteria were applied:

1. Research involving pupae as feed;
2. Research describing the composition of the larval stage;
3. Research published before the year 2000.

As a result, the entire time frame of the systematic review is from 1 January 2000, through 5 May 2021. Additionally, we excluded data from food labeling when SP had been purchased from suppliers as food and only kept original data from the selected trials that were gathered in the research through an analytical method. Furthermore, we did not include information on SP fed a particular diet supplement. The information obtained from the investigations was arranged into pre-defined tables. Three researchers conducted this second stage (L.T., A.S., and S.C.). Information on silkworm strain, feeding (mulberry leaves or artificial diet), and sample processing was provided when available.

Insect-killing techniques, sample storage conditions, and drying technologies used before the analytical stages are among the sample processing variables that may have an impact on compositional results. Another potential source of variation was the analytical approach itself, which varied throughout the articles, particularly for fiber and ash. The existence of experimental replicates and the standard deviation, which were mentioned in this review if they were accessible in the original manuscripts, are additional factors to take into account. The relative standard deviation was estimated in cases when the data were required to be summed up or standardized using the principles of error propagation.

Data about chemical composition were extracted and normalized, if necessary, to be comparable among themselves. This included information about total nitrogen or protein content, ash, lipid or fat content, crude fiber, nitrogen-free extracts (NFE), amino acid (AA) profile, fatty acid (FA) profile, and oligo-elements. All these extracted data were reported in Table 2.

Only a small percentage of the selected papers stated the composition on a wet basis, necessitating the calculation of the composition on a dry matter basis using the moisture values. Therefore, the nutritional values were normalized according to the dry matter content and expressed as a percentage or “g per 100 g of dry matter”. Protein content was also expressed using the nitrogen-to-protein conversion factor suggested by Janssen et al. [16].

To normalize and compare data given in articles included for the final review, amino-acid absolute values for the AA profile were transformed into percentage values (Table 3). The FA profile is shown in Table 4 as a percentage of all FAME (Fatty Acid Methyl Esters). To make data comparison easier, the isomeric forms of the same FA were combined together. The last step was to express the macro and microelements as mg/g on a dry matter basis (Table 5). According to the principles of error propagation, the standard deviation of the data produced by elaboration (addition or normalization of the dry matter) of the original values was determined.

## 3. Results

**Figure 1:**
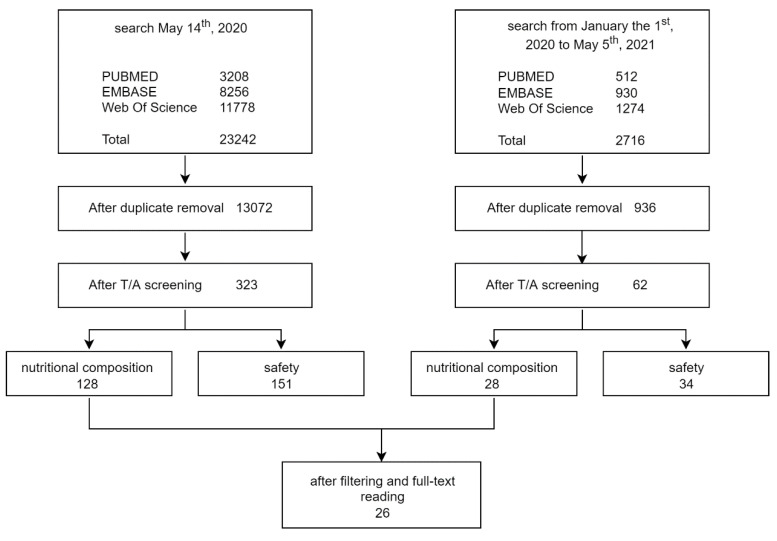
Representation of the procedure used to review and select the studies from which to extract the data for the current systematic review.

After removing duplicates and reading the abstracts of all 14,008 papers that were found using our search parameters and logical operators, 385 of them were retained for additional analysis. Among them, 185 of the chosen studies addressed safety issues, and 156 dealt with chemical composition. As previously said, the former were taken into consideration for a second evaluation of safety issues. Twenty-six papers out of 156 dealing with chemical composition met our inclusion criteria and were utilized to gather data on nutritional information (Figure 1).

### 3.1. Macronutrients and Ash

A total of thirty-three data entries were retrieved from 16 studies reporting at least one nutrient value (Table 2). Two data entries referring to protein values on fresh weight [17] were not considered due to the absence of the moisture value that would have made the two values comparable. The two main types of reported data were “full-fat” SP and “defatted” SP. The values of protein (*n* = 33), lipids (*n* = 26), ash (*n* = 25), nitrogen-free extracts (*n* = 17), and crude fibers (*n*= 16) were reported in the 33 data entries.

An average nitrogen-to-protein conversion factor of 6.25 was used to calculate the protein values in each study. The corrected nitrogen-to-protein conversion factor of 4.76 proposed by Janssen et al. [16] was only adopted by Akande et al. [18] and Birman et al. [19]. The nitrogen-to-protein conversion factor has been “insect adjusted,” and the protein values are as reported in the original studies shown in Table 2. The results discussed below, for simplicity’s sake, apply to protein values obtained using the standard nitrogen conversion factor of 6.25. In three studies including defatted SP, protein levels ranged from 67.5 [20] to 82.9% [21], while residual lipids ranged from less than 0.2 [22] to 4.75% [20].

**Table 2:** Macro nutrient values and main processing variables (g/100 g) retrieved from selected articles. Data were normalized on the dry matter where necessary. * Protein values were originally calculated using Kp = 4.76 as a nitrogen conversion factor. ** the values were indicated in the original paper as “total fiber”.

Reference	Treatment, Strain, Diet				Separation	Protein (Kp = 6.25)	Protein (Kp = 4.76)	Fat	Crude Fiber	NFE (Often Indicates as Carbohydrates in the Original Paper)	Ash
Mishra et al., 2003 [5]	Cocoons were boiled at 100 °C for 30 min. Pupae were taken out and dissected to remove the intestine and waste material. Composition was expressed on wet weight (100 g).				full-fat	34.38 ± 0.25	26.19 ± 0.19	57.64 ± 0.22	0.54 ± 0.00	5.16 ± 0.19	2.27 ± 0.17
Akande et al., 2020 [18]	Edible insects boiled without seasonings were prepared with 100 g of each of the edible insects with 150 mL of potable water and boiled for 15 min. Data are referred to as boiled silkworm pupae without seasoning (SWOS).				full-fat	47.17 ± 1.36	35.92 ± 1.04 *	32.16 ± 0.85	1.68 ± 0.02	28.15 ± 1.23	2.12 ± 0.03
David-Birman et al., 2019 [19]	Finely milled silkworm pupae flour (SWF) was purchased from JR Unique Foods Ltd. (Udon Thani, Thailand).				full-fat	53.05 ± 0.09	40.4 ± 0.07 *	29.56 ± 0.08	n/a	n/a	n/a
Kim et al., 2016 [20]	Dried, untreated SP flour. The whole insects were cleaned with distilled water, sieved, ground using a hammer mill, and passed through a 20-mesh sieve.				full-fat	47.87 ± 1.72	36.46 ± 1.31	30.85 ± 3.81	6.38 ± 0.32 **	n/a	6.96 ± 0.61
			The SP flour was then defatted using hexane		defatted	67.54 ± 0.97	51.44 ± 0.74	4.75 ± 1.79	9.54 ± 0.58 **	n/a	9.54 ± 1.22
			SP defatted using hexane. The insect flour was also acid-hydrolyzed and dried in the dry oven at 70 °C		defatted	73.51 ± 0.74	55.99 ± 0.56	2.05 ± 0.5	3.24 ± 0.19 **	n/a	8.04 ± 0.18
Anootthato et al., 2019 [21]	Frozen silkworm pupae (−20 °C) were thawed at 4 °C overnight and washed three times before blanching at 95 °C for 10 min.				full-fat	56.64 ± 0.42	43.14 ± 0.32	34.07 ± 0.62	n/a	n/a	n/a
			The sample was further dried at 60 °C for 18 h. Dried samples were ground and packed in an aluminum foil bag until use		full-fat	52.69 ± 0.46	40.13 ± 0.35	36.21 ± 0.23	n/a	n/a	n/a
			The sample was further dried at 60 °C for 18 h, ground, and packed in an aluminum foil bag until use. Dried samples were mixed and defatted using 99.8% ethanol (1:3 *w*/*v*) at 50 °C with stirring for 30 min. Then, each sample was filtered. Defatting was carried out twice before drying in the tray dryer at 50 °C for 3 h. Defatted samples were ground to 150 μm before hydrolysis		defatted	82.88 ± 0.5	63.12 ± 0.38	1.47 ± 0.44	n/a	n/a	n/a
Felix et al., 2020 [22]	The silkworm protein concentrate (SPC) used in this study was supplied by FeedStimulants (Amsterdam, The Netherlands).				full-fat	55.43 ± 1.10	42.22 ± 0.84	31.72 ± 0.22	n/a	5.60 ± 1.42	7.24 ± 0.88
			SPC was defatted using n-hexane		defatted	81.14 ± 2.06	61.80 ± 1.57	<0.2	n/a	8.23 ± 2.04	10.63 ± 1.27
Akande et al., 2020 [23]	Freshly harvested mulberry silkworm pupae (SWP) were stifled in an oven at 93 °C for 1 h, cut out of their cocoons and dried in a hot air oven (Gen. Lab. oven) at 40 °C for 8 h. The dried SWP was milled into flour using an electric blender (Binatone, Model No. 51-777) and kept airtight. The data are referred to as silkworm pupae powder (SWP).				full-fat	60.7 ± 0.2	46.23 ± 0.15	23.5 ± 0.21	1.1 ± 0.16	11.3 ± 0.42	0.9 ± 0.56
Kim et al., 2016 [24]	The dried edible insects were purchased from three vendors located in Chungnam, Korea. Nutrients were originally expressed as g/kg dry weight.				full-fat	52.58 ± 0.36	40.04 ± 0.27	19.21 ± 0.34	n/a	23.41 ± 0.01	4.8 ± 0.71
Kuntadi et al., 2018 [25]	The SP were oven dried at 60–70 °C for 12–24 h and then ground.				full-fat	60.03	45.72	29.47	n/a	0.92	5.79
Lamberti et al., 2019 [26]	SP were collected 7 days after reaching the cocoon stage. Pupae were lyophilized and stored at −80 °C.	male silkworm reared on an artificial diet			full-fat	61.45	46.80	n/a	n/a	n/a	5.68
		male silkworm reared on leaf			full-fat	55.02	41.90	n/a	n/a	n/a	4.62
		female silkworm reared on an artificial diet			full-fat	72.36	55.11	n/a	n/a	n/a	5.46
		female silkworm reared on leaf			full-fat	56.29	42.87	n/a	n/a	n/a	5.02
Rodriguez-Ortega et al., 2016 [27]	Dried pupae. Larvae reared on mulberry tree leaves.				full-fat	64.31 ± 0.00 (true protein = 46.87 ± 0.00)	48.98 ± 0.00	20.63 ± 0.105	4.89 ± 0.107	3.92	6.25 ± 0.017
Tomotake et al., 2010 [28]	SP were purchased from Nishiki Food Ltd. (Nagano, Japan). SP were lyophilized followed by grinding into a fine powder.				full-fat	55.60	42.34.00	32.20	n/a	n/a	n/a
Pereira et al., 2003 [29]	Silkworm pupae fed with younger leaves of mulberry tree, and dried at 130 °C, until 4–5% of moisture.				full-fat	51.1 ± 1.8	38.92 ± 1.37	34.4 ± 0.8	n/a	n/a	3.64 ± 0.09
Ghosh et al., 2020 [30]	Pupae of *B. mori* were ground in a mortar and pestle and centrifuged adding some distilled water. Then the liquid centrifugate was spray-dried and ground into fine powdered material.				full-fat	55.87 ± 1.16	42.55 ± 0.88	23.45 ± 0.28	3.77 ± 1.16	1.76 ± 1.19	5.38 ± 0.07
Mishyna et al., 2020 [31]	Insects were stored at −20 °C prior to grinding or drying. After drying, the insects were ground in a food blender and stored in a double plastic bag at −20 °C prior to use, but no longer than 1 month.			freeze-dried	full-fat	60.29 ± 0.38	45.91 ± 0.29	n/a	n/a	n/a	n/a
				oven-dried	full-fat	59.33 ± 0.12	45.19 ± 0.09	n/a	n/a	n/a	n/a
				microwave-dried	full-fat	61.57 ± 0.21	46.89 ± 0.16	n/a	n/a	n/a	n/a
Hirunyophat et al., 2021 [32]	Frozen and stored at −18 °C.			Nangnoi (NN) strain	full-fat	54.83 ± 0.43	41.76 ± 0.33	23.61 ± 0.56	2.67 ± 0.06	14.99 ± 1.05	3.99 ± 0.11
				Siwtui (ST) strain	full-fat	55.39 ± 0.19	42.19 ± 0.14	21.26 ± 0.92	2.91 ± 0.92	17.45 ± 0.83	3.12 ± 0.06
				Luang Saraburi (LS) strain	full-fat	50.36 ± 0.21	38.35 ± 0.16	27.59 ± 0.43	3.29 ± 0.07	13.42 ± 0.43	5.41 ± 0.09
				Ubon Ratchathani 60–35 (U 60–35) strain	full-fat	48.31 ± 0.55	36.79 ± 0.42	29.93 ± 0.85	2.81 ± 0.03	14.87 ± 0.08	4.23 ± 0.47
	Frozen and stored at −18 °C, thawed at 5 °C for 12 h, and dried at 60 °C for 16 h; ground to pass through 177-micron mesh sieve; stored at −18 °C; composition dry weight.			Nangnoi (NN) strain	full-fat	52.94 ± 0.11	40.32 ± 0.08	25.53 ± 0.01	4.78 ± 0.06	12.00 ± 0.09	4.69 ± 0.05
				Siwtui (ST) strain	full-fat	56.00 ± 0.4	42.65 ± 0.3	23.95 ± 0.15	5.58 ± 0.09	10.78 ± 0.7	3.94 ± 0.02
				Luang Saraburi (LS) strain	full-fat	49.19 ± 0.26	37.46 ± 0.2	30.47 ± 0.33	5.46 ± 0.14	9.93 ± 0.76	4.73 ± 0.06
				Ubon Ratchathani 60–35 (U 60–35) strain	full-fat	46.22 ± 0.15	35.2 ± 0.11	34.69 ± 0.27	4.92 ± 0.19	9.82 ± 0.03	4.25 ± 0.02

The principal method utilized to remove lipids from the samples was chemical extraction, utilizing ethanol or hexane as the organic solvent. Full-fat SP was the subject of 16 investigations, yielding a total of 31 data entries. Protein levels in full-fat SP ranged from 34.4 [5] to 72.4% [26]. The lowest value was reported by Mishra et al. [5] and related to SP that had been boiled, dissected, and had the inner content removed. Lamberti et al. [26] discovered female pupae raised on an artificial diet displayed the greatest protein value. Rodriguez-Ortega et al. [27] determined that the highest protein value was 64.3% when only pupae raised on mulberry leaves were used. Lamberti et al. [26] also investigated the protein differences between males and females, and the results highlighted the highest values for females. When microwave-dried SP was compared to oven-dried SP, freeze-dried SP, and other types of dried SP, Mishyna et al. [31] discovered that microwave-dried SP had a considerably higher protein value.

The total lipid content ranged from 19.2 [24] to 57.6% [5] and was available for 22 data entries pertaining to full-fat SP. The majority of the research that dealt with lipid extraction methods used standard laboratory extraction procedures using n-hexane, chloroform, methanol, and ethanol as the extraction solvents.

It should be noted that the study by Wei et al., was the only one to investigate supercritical CO_2_ as the extraction solvent [33], while Hu et al. employed a microwave-assisted extraction using a mixture of ethanol and n-hexane as the extraction solvent [4]. The crude fiber content of full-fat pupae ranged from 0.54 [5] to 6.38% [24]. For this nutrient, only 14 data entries were available. Nitrogen-free extract content was provided for 17 data entries and ranged from 0.92% [25] to 28.2% [18]. In most of the studies, NFE was referred to as “carbohydrate” and calculated by difference. Ash content ranged from 0.9 [23] to 7.94% [22] for 22 data entries.

### 3.2. Amino Acid Profile

Table 3 presents a total of 11 data entries from six original investigations about the AA profile. The relative abundance of each AA was given as a percentage of the total amount, albeit the values were biased because only three studies quantified all the AA. A complete comparison was impossible since two studies failed to provide the tryptophan value and one failed to provide tryptophan, cysteine, and methionine values [21,30,31].

We further assumed that asparagine and glutamine values were included in aspartic and glutamic acid values, respectively, as a result of the widespread AA measurement methods [34]. However, research by Kwon et al. [35] emphasized how the value of certain AA varied over the course of pupae development; for instance, histidine values declined with pupal age while threonine and serine values increased. There are a few differences between the AA reported in this study for pupae that are 12–13 days old (D12–13) and for pupae that are 13 days old (D–13), but the comparison was not possible since the size and variability of the samples are unknown.

### 3.3. Fatty Acid Profile

Seventeen data points about the FA profiles were taken from nine papers and reported in Table 4. The single FA appears to be influenced by a number of variables, including SP strain, nutrition, pupal age, sex, drying and extraction procedure, and analytical methodologies. According to research by Kwon et al. [35] on the influence of pupal age on the composition of fatty acids, linoleic acid was the most variable FA between days 10 and 13, when its relative abundance varied from 5.7 to 8.7% total FAME.

**Table 3:** Amino acid composition. When available in the original data, values given as a percentage of the total AA along with their respective SD.

Reference		Khoeler et al., 2019 [17]		Akande et al., 2020 [18]	Anootthato et al., 2019 [21]	Tomotake et al., 2010 [28]	Kwon et al., 2012 [36]				Shi et al., 2018 [37]	
**Treatment**		**SH (ready-to-eat, deep-fried in cooking oil. Samples were frozen and freeze-dried for 24 h, powdered and conserved at −80 °C)**	**SM (non-fried snack, cooked using steam and hot air. Samples were frozen and freeze-dried for 24 h, powdered and conserved at −80 °C)**	**SP boiled without seasonings, blended**	**Frozen SP were thawed at 4 °C overnight and washed three times before blanching at 95 °C for 10 min. Then, the sample was dried in a tray dryer at 60 °C for 18 h**	**SP were lyophilized, followed by grinding into a powder**	**Frozen SP were extracted and then the protein extract was hydrolyzed with hydrochloric acid at 110 °C for 24 h**				**SP protein hydrolysate, obtained through enzymatic hydrolysis**	
							**Pupal age: Day 12–13**	**Pupal age: Day D7**	**Pupal age: Day D10**	**Pupal age: Day D13**	**Spray dried**	**Freeze dried**
Amino acid	Val	5.95	5.99	4.94 ± 0.04	6.18 ± 0.12	5.53	5.7	4.2	4	2.8	5.27	4.86
	Thr	4.82	4.72	4.62 ± 0.06	5.08 ± 0.07	4.59	3.3	4.6	8.5	9.7	3.89	5.19
	Ile	4.16	4.35	4.92 ± 0.09	4.84 ± 0.10	4	2.7	2.3	1.8	2.1	4.27	2.61
	Leu	7.44	7.39	8.16 ± 0.07	8.16 ± 0.07	7.29	3.5	2.4	2.1	2.5	7.07	6.04
	Lys	7.4	7.19	9.00 ± 0.05	9.09 ± 0.21	7.18	4.5	2.9	3.1	3.9	7.78	7.93
	Met	3.37	4.01	2.28 ± 0.22	-	4	5	6.5	6.4	5	2.42	2.3
	Phe	5.04	5.71	4.21 ± 0.07	5.25 ± 0.02	5.41	3	2.6	1.4	1.6	7.2	7.33
	His	2.98	3.04	2.63 ± 0.06	3.25 ± 0.02	3.18	14.6	25.1	16	13.8	3.16	3.66
	Trp	1.49	1.55	1.28 ± 0.06	-	1.76	-	-	-	-	-	-
	Glu	10.99	11.31	17.02 ± 0.46	13.58 ± 0.21	11.18	18.3	18.9	21.5	18.1	12.44	12.68
	Gln	-	-	-	-	-	-	-	-	-	-	-
	Gly	4.6	4.18	5.16 ± 0.04	4.51 ± 0.05	4.24	7.3	5.2	7	6.1	8.73	10.09
	Ala	5.47	4.85	7.85 ± 0.04	5.80 ± 0.07	4.59	10.2	6.4	3.8	5.1	5.14	4.77
	Ser	4.95	4.61	4.63 ± 0.09	5.30 ± 0.10	4.35	5.2	3.4	9	10.6	3.78	3.1
	Pro	4.55	4.09	3.61 ± 0.05	4.87 ± 0.10	8.24	4.1	6.1	4.4	3.4	9.58	9.76
	Asp	11.69	11.48	8.57 ± 0.04	-	10.71	1.5	1.5	2.2	6	11.23	11.04
	Asn	-	-	-	12.12 ± 0.31	-	-	-	-	-	-	-
	Arg	5.47	5.77	6.62 ± 0.06	6.61 ± 0.10	5.53	3.6	1.3	1.5	2.5	3.25	3.68
	Tyr	8.23	8.46	2.62 ± 0.41	5.35 ± 0.14	6.59	6.2	6.2	6.8	6.7	3.79	4.09
	Cys	1.4	1.31	1.90 ± 0.05	-	1.65	1.5	0.2	0.6	0.4	1	0.88

**Table 4:** Fatty acid profile (% total FAME) of selected studies and data.

Reference	Treatment		C12:0	C14:0	C15:0	C15:1	C16:0	C16:1, C16:1n-7	C16:2n-6	C16:3n-3	C17:0	C17:1	C18:0	C18:1, C18:1n-7, C18:1n-9	C18:2, C18:2(n-6)	C18:3, C18:3(n-3)	C20:0	C20:1, C20:1n-9	C20:3 (and C20:3n-6, C20:3n-3)	C20:4n-6	C20:5, C20:5n-3	C22:0	C24:0	others
Hu et al., 2017 [4]	BMP were dried in a vacuum drier at 70 °C for 24h to a moisture content of <5% and ground into a fine powder. The powder was sieved and stored at 4 °C.	Microwave assisted extraction, using a mixed solvent consisting of ethanol and n-hexane (1:1, *v*/*v*)	-	0.18 ± 0	-	-	23.18 ± 0.52	1.07 ± 0.09	-	-	0.15 ± 0	0.1 ± 0	4.69 ± 0.17	28.32 ± 0.63	3.88 ± 0.13	38.25 ± 0.75	0.16	-	-	-	-	-	-	-
		Soxhlet extraction using n-hexane, at 80 °C for 6 h. The oil was dried at 100 °C ± 5 °C for 15 min. in a drying oven	-	0.19 ± 0	-	-	23.04 ± 0.58	1.05 ± 0.07	-	-	0.17 ± 0	nd ± nd	4.68 ± 0.19	28.15 ± 0.54	3.85 ± 0.54	38.06 ± 0.68	0.16	-	-	-	-	-	-	-
Tomotake et al., 2010 [28]	Silkworm pupae were lyophilized, followed by grinding into a powder. Lipid content was determined according to the methods of AOAC.		-	0.1	-	-	24.2	1.7	-	-	-	-	4.5	26	7.3	36.3	-	-	-	-	-	-	-	-
Pereira et al., 2003 [29]	Chrysalis (worm) toast (originated from the silkworm pupae fed with younger mulberry tree leaves and dried at 130 °C until 4–5% of moisture). Lipids were chemically extracted using chloroform-methanol.		-	0.164 ± 0.02	-	-	24.6 ± 2.1	0.656 ± 0.04	-	-	0.192 ± 0.06	-	7.56 ± 1.54	34.8 ± 3.3	7.03 ± 1.08	24.4 ± 6.7	-	-	0.275 ± 0.1	0.334 ± 0.08	-	-	-	-
Wei et al., 2009 [33]	Silkworm pupae were vacuum dried at 60 °C to a moisture content of <5%, then finely ground to powder, and finally oil was extracted by supercritical CO_2_ extraction.		-	-	-	-	21.77	-	-	-	-	-	7.02	33.26	7.12	27.99	-	-	-	-	-	-	-	2.84
Kwon et al., 2012 [35]	The frozen silkworm pupae were ground, blended, mixed with ethanol and stirred. The sample was filtered, and the particles were extracted with ethyl acetate. The extracts were vacuum-concentrated and dissolved in hexane. Activated carbon was added, and the mixture was heated for 1 h and then cooled and filtered before vacuum-concentration.	Pupal period days 12–13	-	-	-	-	19.7	2.5	-	-	-	-	8.6	19.9	7.4	41.6	-	-	-	-	0.3	-	-	-
		Pupae at day 7	-	-	-	-	22.6	2	-	-	-	-	9.6	21.1	8.2	36.4	-	-	-	-	-	-	-	-
		Pupae at day 10	-	-	-	-	21	1.7	-	-	-	-	8.3	24.3	5.7	38.9	-	-	-	-	0.1	-	-	-
		Pupae at day 13	-	-	-	-	20	1.8	-	-	-	-	8.8	21.8	8.7	38.6	-	-	-	-	0.3	-	-	-
Chieco et al., 2019 [37]	Cocoons were dried for 3 days at 60 °C. The pupae were extracted and stored at −80 °C. Frozen pupae were milled to a fine powder. Lipids were chemically extracted using a chloroform/methanol solution according to the Folch’s method.	White polyhibrid on artificial diet	-	-	-	-	29.2 ± 0.4	1.5 ± 0	-	-	-	-	10.9 ± 0.1	35.1 ± 0.3	11.4 ± 0.5	11.7 ± 0.3	-	-	-	-	-	-	-	-
		Golden nistari on artificial diet	-	-	-	-	25.1 ± 0.3	1.5 ± 0	-	-	-	-	9.3 ± 0.2	39.8 ± 0.3	11.8 ± 0.1	12.3 ± 0.2	-	-	-	-	-	-	-	-
		White polyhibrid on mulberry leaf of the Florio cultivar	-	-	-	-	25.2 ± 0.7	0.8 ± 0.1	-	-	-	-	7.2 ± 0.2	35.2 ± 0.3	7.1 ± 0.4	24.3 ± 0.2	-	-	-	-	-	-	-	-
		Golden nistari on mulberry leaf of the Florio cultivar	-	-	-	-	21.6 ± 0.4	1.4 ± 0	-	-	-	-	4.8 ± 0.1	32.3 ± 0.1	10.4 ± 0.2	29.3 ± 0.2	-	-	-	-	-	-	-	-
Tong et al., 2011 [38]	Silkworms were directly frozen on the third day of the 5th instar and then lyophilized. Lipid content was measured using Soxhlet extraction.		-	-	0.15	-	16.03	0.39	-	-	-	-	9.45	28.12	12.24	31.91	0.7	0.15	-	-	-	0.7	0.15	-
Usub et al., 2008 [39]	Sun-dried pupae. Five grams were grounded and the lipid extracted with 50 mL of chloroform-methanol (2:1, *v*/*v*) containing 10 mg L^−1^ of butylated hydroxytoluene and 0.1 mg L^−1^ of tricosanoic acid.		0.1 ± 0	0.1 ± 0	0.1 ± 0	0.1 ± 0	17 ± 0.1	0.6 ± 0	-	-	0.2 ± 0.1	-	6.2 ± 0	20.4 ± 0.1	8.8 ± 0.1	45.6 ± 0.2	0.4 ± 0	0.1 ± 0	0.1 ± 0	0.1 ± 0	-	-	-	-
	Solar-tunnel dried pupae. Five grams were ground and the lipid extracted with 50 mL of chloroform-methanol (2:1, *v*/*v*) containing 10 mg L^−1^ of butylated hydroxytoluene and 0.1 mg L^−1^ of tricosanoic acid.		0.1 ± 0	0.1 ± 0	0.1 ± 0	0.1 ± 0	17.5 ± 0.6	0.6 ± 0	-	-	0.2 ± 0	-	6.2 ± 0.1	21 ± 0.2	8.8 ± 0.1	44.6 ± 0.5	0.4 ± 0	0.1 ± 0	0.1 ± 0	0.1 ± 0	-	-	-	-
Wang et al., 2020 [39]	Silkworm pupae were dried in a vacuum at 60 °C for 24 h to a moisture content of <5% and then slightly ground and sieved through a 60-mesh sieve. Lipid extraction was conducted using the Folch and Sloane method.		-	-	-	-	22.04 ± 0.49	0.92 ± 0.01	-	-	-	-	6.84 ± 0.23	33.91 ± 0.08	5.48 ± 0.14	30.81 ± 0.21	-	-	-	-	-	-	-	-
Yu et al., 2018 [40]	Pupae were frozen in liquid nitrogen and stored at −80 °C before analysis. Pupae were homogenized and oil was chemically extracted using hexane and boron trifluoride-methanol solution.	Three-day old silkworm pupae, strain Dazao, fed with an artificial diet (Silkmate 2S), Table 2 in the original paper	-	-	-	-	16.6 ± 2.38	0.51 ± 0.04	0.55 ± 0	0.38 ± 0	-	-	11.26 ± 0.7	28.53 ± 1.83	20.54 ± 1.68	21.13 ± 1.7	0.51 ± 0.12	-	-	-	-	-	-	-
		Three-day-old silkworm pupae, strain Dazao, fed with an artificial diet (Silkmate 2S), Table 3 in the original paper	-	-	-	-	15.61 ± 1.89	0.46 ± 0.09	0.46 ± 0.1	0.23 ± 0.17	-	-	12.87 ± 1.4	26.14 ± 1.41	22.24 ± 1.25	21.31 ± 1.21	0.67 ± 0.2	-	-	-	-	-	-	-
		Three-day-old silkworm pupae, strain Dazao, female	-	-	-	-	17.37 ± 0.53	0.53 ± 0.06	0.48 ± 0.06	0.33 ± 0.05	-	-	12.55 ± 1.32	26.63 ± 1.4	21.32 ± 0.53	20.26 ± 0.59	0.53 ± 0.19	-	-	-	-	-	-	-
		Three-day-old silkworm pupae, strain Dazao, male	-	-	-	-	14.34 ± 1.19	0.45 ± 0.08	0.55 ± 0.06	0.36 ± 0.05	-	-	12.54 ± 1.46	25.38 ± 1.72	23.11 ± 1.09	22.57 ± 0.92	0.71 ± 0.11	-	-	-	-	-	-	-
		Three-day-old silkworm pupae, strain 305, female	-	-	-	-	18.44 ± 1.59	1.19 ± 0.57	0.53 ± 0.03	0.29 ± 0.15	-	-	12.93 ± 2.14	28.23 ± 2.08	18.72 ± 1.05	19.14 ± 1.3	0.53 ± 0.18	-	-	-	-	-	-	-
		Three-day-old silkworm pupae, strain 305, male	-	-	-	-	15.23 ± 0.36	1.16 ± 0.58	0.57 ± 0.08	0.38 ± 0.06	-	-	13.12 ± 0.96	25.97 ± 1.57	21.2 ± 1.3	21.54 ± 0.64	0.83 ± 0.38	-	-	-	-	-	-	-

The two extraction techniques—microwave and Soxhlet—used in the study by Hu et al. [4] and the two drying techniques—sun drying and solar tunnel drying—tested by Usub et al. [39] appear to have minimal impact on the final FA profile. Additionally, the FA profile is significantly impacted by the use of an artificial diet during the larval stage. The use of an artificial diet, as well as the usage of various silkworm strains, were both examined in the study by Chieco et al. [37]. The former had a significant impact on the SP’s FA composition by raising stearic acid and lowering alfa-linolenic acid. Regarding the silkworm strain, it was shown that the pupae of polyhybrid and Nistari (a tropical pure strain) show notable differences in FA profile, with the Nistari strain having the largest concentration of linoleic and linolenic acids.

Two distinct strains were raised on an artificial diet in the study by Yu et al. [40] and the FA composition was assessed in relation to strain and sex. The findings suggest that higher levels of unsaturated FA (UFA) characterize female pupae, and they probably account for the largest quantities of energy devoted to oviposition. According to this study, the investigated strains were similar in terms of FA profile.

When comparing the findings obtained by Yu et al. [40], to those from other research where silkworms were grown on mulberry leaves, it was found that the concentration of the palmitic (C16:0), linolenic (C18:3), and linoleic (C18:2) acids decreased while the content of linoleic (C18:2) increased. All the investigations came to the same conclusion about the five primary FAs that characterized the SP. In fact, the acids palmitic (C16:0), stearic (C18:0), oleic (C18:1), linoleic (C18:2), and alfa-linolenic (C18:3) make up between 97.1 percent [41] and 99.1 percent [39] of total FAME. Among the five FAs, linoleic and alfa-linolenic acids are essential FAs. Their amount fluctuates between 23.1% and 54.4% of total FAME, with pupae grown on an artificial diet having the lowest value [37].

Furthermore, palmitoleic acid (C16:1 n-7) contributes a small but consistent amount to total FA quantity (0.51%–2.00% total FAME). A few FAs were found in single studies. Among unsaturated FA, C16:2n-6 and C16:3n-3 were identified only by Yu et al. [40]. Considering saturated FA, C22:0, and C24:0 were identified only by Tong et al. [38], while C12:0 appears only in the study by Usub et al. [39]. The percentage of total saturated FA (SFA) in total FAME ranges from 24.1 to 40.1%. In particular, the highest values were reached by those silkworms reared on an artificial diet [37].

### 3.4. Mineral Content

From six studies, a total of seven data entries about mineral content were accumulated and presented in Table 5. Iron, zinc, manganese, magnesium, and calcium were among the minerals found and quantified in the majority of the publications. It is important to note that the magnitudes of the minerals identified in the study by Akande et al. [23] differed greatly from the other studies. This difference could be because pupae were boiled before analysis, and leaking mineral components might have affected the results.

Iron concentrations ranged from 2.83 to 4.95 mg/100 g, calcium from 92.0 to 181 mg/100 g, manganese from 1.08 to 2.30 mg/100 g, zinc from 1.39 to 24.4 mg/100 g, and magnesium from 89 to 280 mg/100 g, excluding the values found in the study by Akande et al. [23]. Other minerals were quantified in a few studies and included sodium, which ranged from 29.6 [29] to 363 mg/100 g [17].

Three investigations showed potassium content, which ranged from 477 to 672 mg/100 g. Table 5 lists additional minerals that were mentioned in the selected papers.

**Table 5:** The mineral content of selected studies and data. Akande et al., 2020, originally expressed it as ppm. Kim et al., 2016, originally expressed it as mg/kg. Rodriguez-Ortega et al. 2016, originally expressed it as a percentage of dry matter. Köhler et al., 2019, expressed it as mg/100g of edible portion (EP).

Ref.		Köhler et al., 2019 [17]		Akande et al., 2020 [18]	David-Birman et al., 2019 [19]	Kim et al., 2016 [24]	Kuntadi et al., 2018 [25]	Rodriguez-Ortega et al., 2016 [27]	Pereira et al., 2003 [29]
		mg/100 g, (Edible Portion)		M ± SD; mg/100 g	M ± SD; mg/100 g (dry weight)	M ± SD; mg/100 g (dry weight)	mg/100 g (dry weight)	Mean ± SD; mg/100 g (dry weight)	M ± SD; mg/100 g (dry weight)
		Street Hawker	Supermarket						
Minerals	Cr	-	-	-	N/D	-	-	-	0.253 ± 0.023
	Hg	<0.005	<0.005	-	N/D	-	-	-	-
	Pb	0.0138	0.0044	-	N/D	-	-	-	-
	Cd	<0.005	<0.005	-	N/D	-	-	-	-
	As	0.0432	0.0165	-	-	-	-	-	-
	Se	0.0534	0.2285	-	-	-	-	-	-
	Mo	0.0151	0.0516	-	-	0.02 ± 0.00	-	-	-
	B	-	-	-	-	1.46 ± 0.42	-	-	-
	Cu	0.711	0.943	-	-	0.94 ± 0.01	-	-	1.52 ± 0.07
	Na	128.4	362.6	-	41.86 ± 3.45	-	-	-	29.60 ± 2.10
	K	492.9	672	-	513.8 ± 1.13	-	-	-	477 ± 16
	Mg	157.7	174.7	-	280.8 ± 1.13	252.29 ± 10.26	-	-	89 ± 0
	Fe	2.83	3.18	0.142 ± 0.063	N/D	4.95 ± 0.05	3.54	-	3.00 ± 0.88
	Zn	9.8	15.4	0.203 ± 0.004	1.39 ± 0.07	14.70 ± 1.45	-	-	24.40 ± 0.96
	Mn	1.26	1.86	0.021 ± 0.021	1.08 ± 0.05	1.68 ± 0.06	-	-	2.30 ± 0.17
	Ca	92.2	107.6	0.249 ± 0.013	137.01 ± 1.34	98.72 ± 0.00	29.17	760 ± 0	181 ± 9
	P	492.9	672	5.57 ± 0.144	-	870.92 ± 15.77	-	720 ± 0	-

M ± SD = mean ± standard deviation; N/D = not detected.

## 4. Discussion

Protein content was the nutritional value that was most frequently reported, which is indicative of the fact that silkworm pupae were primarily seen as a promising protein source. The removal of the inner material, an unusual method in insect processing that could have altered the nutrient content of SP, may have caused the high value of lipids and low value of protein discovered by Mishra et al. [5]. Most of the protein values for full-fat SP were between 45 and 60% (percentage of the dry matter), which was comparable to other raised insects such as *Tenebrio molitor* and *Grillus bimaculatus* (53.2 and 58.3%, respectively) [42] and chicken eggs (54.1%) [43].

For the majority (86%) of the samples, the full-fat SP’s total lipid content ranges from 20 to 35%. These figures could be in line with those discovered for chicken (34.8%), swine (26.3%), tuna (24.9%), and *T. molitor* (34.5%) expressed on a dry matter basis [42,43]. It should be noted that when comparing nutritional data for insects to that of other traditional animal sources, the nutritional content of insects is expressed as referring to the full organism, as opposed to traditional foods of animal origin, where only a portion of the animal is consumed.

A nitrogen-to-protein conversion factor (Kp) is frequently used to determine the protein content from the total nitrogen determined using analytical techniques. Most of the studies used a Kp of 6.25, whereas only two reported an insect corrected Kp of 4.76. The 6.25 conversion factor is the most commonly used for protein estimation in meat. However, it is based on the assumption that all of the nitrogen found in a food matrix comes from protein and that nitrogen accounts for 16 percent of protein [44]. This could result in an overestimation of protein content, particularly in insects that are characterized by the presence of chitin. As a result, Janssen et al. [16] proposed using a Kp conversion factor of 4.76 based on the quantification of non-protein nitrogen (i.e., chitin and chitosan, nucleic acids, and urea) in three insect species: *Hermetia illucens*, *Tenebrio molitor*, and *Alphitobius diaperinus*. Moreover, because NFE is usually calculated by difference, an incorrect estimation of protein content could lead to an incorrect estimation of NFE content. For these reasons, and in the absence of a specific Kp for SP, we agree to use the 4.76 Kp value proposed by Janssen et al. [16]. The presence of all essential AA, characterized SP’s amino acid content, but the variability of the retrieved data precludes further considerations. Kwon et al. [35] found variation in AA profile as a function of pupal age, which should be taken into account during the setup of rearing and further processing of SP in relation to the desired AA profile in the final product.

The main issue with the AA dataset was that only two of the six studies performed replications of their analyses, making data comparison even more difficult. Despite the variability of FA identified in the selected studies, five main FAs (palmitic, stearic, oleic, linoleic, and alfa-linolenic acid) characterized SP oil, making it a good source of UFA, whose content ranges between 59.7 and 75.8%, with the lowest values (from 59.7 to 65.4%) in SP reared on an artificial diet [37], which also have the highest SFA.

The majority of the UFA retrieved values were between 65 and 75%; however, the proportion of monounsaturated FA (MUFA) and polyunsaturated FA (PUFA) that contributes to UFA content varies across papers. The SP reared on an artificial diet had the highest MUFA values as well as the lowest PUFA values [37], implying that the use of an artificial diet could change the proportion of FA classes, reducing PUFA in favor of MUFA and SFA. Nonetheless, the SP reared on an artificial diet by Yu et al. [40] had PUFA and MUFA values comparable to all other SP reared on mulberry leaves. The findings of Chieco et al. [37] and Yu et al. [40] indicate that the use of an artificial diet has a significant impact on the SP FA profile.

Domesticated silkworms are strictly monophagous insects that feed primarily on fresh mulberry leaves (*Morus alba* L.) but can also be reared on artificial diets [44]. In artificial diets, mulberry leaf powder is typically mixed with plant-based meals (soybean, wheat, or corn meals), vitamins, antioxidants, and inorganic salts [44,45]. As previously demonstrated [46], the type and proportion of the different plant matrices included in the diet determine the FA profile of the diet and are likely to influence the SP FA profile.

When we looked at the proportion of n-3 and n-6 FA in SP fed mulberry leaves versus an artificial diet, we discovered that SP fed mulberry leaves have an n-6/n-3 FA ratio ranging from 2.80:1 to 9.88:1, whereas those fed an artificial diet have an n-6/n-3 FA ratio ranging from 0.96:1 to 1.05:1. This is primarily due to the comparable amounts of linoleic and alfa-linolenic acid and a much lower amount of n-3 FAs in the artificial diet fed to SP, indicating that the diet appears to be the main variable influencing the SP’s FA profile.

Since they are not synthesizable by the human body, n-3 and n-6 FA play an important role in the human diet and are thus considered essential FA. Furthermore, their ratio influences body fat metabolism and systemic inflammation, with an increase in the n-6/n-3 ratio associated with an increased risk of obesity, atherosclerosis, and diabetes [47,48]. As a result, an n-6/n-3 FA ratio of 1:1 to 5:1 is thought to be optimal for human health [49]. The average Western diet has an n-6/n-3 ratio of 15.0:1 to 16.7:1, so it would be preferable to include food supplements or nutraceuticals with a lower n-6/n-3 ratio in the Western diet. Furthermore, lowering the n-6/n-3 ratio in broiler diets has been shown to improve growth performance and immune response in these birds [50]. In the case of other farmed animals’ meat, n-6/n-3 values of 1.3, 2.1, 7.2, and 13.7 were found in the muscle tissues of sheep, cattle, pigs, and chickens, respectively [51,52]. The n-6/n-3 ratio is always lower in marine species such as herring, tuna, pollock, salmon, and rainbow trout because their diet is based on marine phytoplankton [53]. The values discovered for mulberry leaves fed SP are thus comparable to those found in sheep, cattle, and pigs.

The linoleic and alfa-linolenic acid content of mulberry leaves fed with SP varied greatly, but it is unclear which variable could explain these results. Consequently, in order to achieve a better optimal n-6/n-3 ratio, we must identify the most relevant variables and their cause-effect relationships.

In terms of the mineral content of SP, the available literature indicates that mulberry is important in determining the mineral content of silkworms, which bioaccumulate minerals through nutrition. The high variability in SP mineral content observed could thus be linked to mineral availability in the soil where the mulberry tree grows. Mineral uptake in the mulberry tree may also vary in response to heavy metal contamination in the soil [54]. Mulberry cultivar and harvest time are also important factors in mineral accumulation [55].

The high sodium value discovered by Kohler et al. [17] was found in a supermarket sample, and it cannot be excluded that it was due to the addition of additives. The *B. mori* strain used is another variable that influences the SP nutrient composition, as demonstrated by Hirunyophat et al. [32]. This information is frequently missing, except in papers in which the strain was one of the primary factors investigated. However, when not reported, the analysis would have most likely been performed on hybrid silkworms, which are the most common type in experimental and production settings. Although the impact of several intrinsic factors on the nutritional value of SP has been investigated, it is not always clear how the analytical methods were implemented, and especially if and how the analyzed pupae were preprocessed.

When silkworms were not reared in a laboratory or under controlled conditions, the process they went through to reach the pupal stage was sometimes unclear. This is especially true for SP recovered from silk reeling industries (by-product pupae), for which precise data on industrial processing, storage time, and temperature were not available [56]. Silkworm pupae from reeling industries have often been dried and boiled, which may have altered the nutritional content. Furthermore, once recovered from the reeling plants, they were most likely dried and stored under highly oxidative conditions. These factors may have a significant impact on the composition and must be considered if the pupae’s final destination is the feed or food market.

Finally, as reported by Kwon et al. [35], pupal age may also play a role in determining the composition. It is worth noting that because only 64% of the data has a standard deviation or other measures of variation associated with mean values, a large portion of them are statistically weak and difficult to compare.

## 5. Conclusions

The literature on SP as food has increased recently, and the topic is expected to be investigated further, given the growing interest in insects as food. Our systematic review, which focuses on the nutrient composition of SP, highlights the wide range of data obtained. On the one hand, the variables involved in sample preparation and analysis are frequently well described in the studies; on the other hand, variables such as the mulberry cultivar used to feed the larvae, silkworm strain, and rearing environmental conditions are rarely reported. When SP was obtained from the silk reeling industry, pre-treatments, and storage conditions were also poorly described or unknown. Furthermore, studies reported data in various units of measurement or formats, making quick comparison difficult.

For all these reasons, new studies with more detailed and better described analytical methodologies, normalized data on a dry matter basis, and discussion about those values that significantly differ from previously reported literature are urgently needed. It is recommended that standard methods from other product sectors be established or adapted to analyze the nutritional values of edible insects for the food market (and feed as well) and that a reference benchmark for the most commonly used analytical procedures be established.

In terms of nutritional composition, SP is primarily distinguished by its protein content, which is the most commonly reported variable in papers. The wide range of values found in the literature for protein content and AA profile requires further study, as do the variables that influence those values. When it comes to the FA profile, it is worth noting that the available literature agrees on identifying five major FAs that account for more than 97.1% of the total SP lipid content. The balance of these five main FAs has been shown to be influenced by diet and pupal age, but the sum of these five FAs is very consistent regardless of upstream conditions.

## Figures and Tables

**Table 1:** Keywords employed to retrieve relevant records reporting data on silkworm food safety and composition. The first keyword refers to the species and any alternate names it may have. The second keyword refers to all the terms related to nutritional composition that have been investigated in this review, while the last keyword refers to food safety terms. The first keyword was searched in conjunction with the second or third, linking them with logical operators.

Keywords (Title/Abstract)				
Bombyx OR Silkworm OR “silk worms” OR silkmoth OR “silk moths”	AND	nutrition OR composition OR centesimal OR nutrient OR nutrients OR protein OR proteins peptide OR peptides OR aminoacid OR aminoacids OR “amino acid” OR “amino acids” OR acid OR acids OR polypeptide OR polypeptides OR fat OR fats OR lipid OR lipids OR “fatty acid” OR “fatty acids” OR “fatty alcohols” OR sugar OR sugars OR carbohydrate OR carbohydrates OR disaccharide OR disaccharides OR monosaccharide OR monosaccharides OR polysaccharide OR polysaccharides OR ash OR mineral OR minerals OR macronutrient OR macronutrients OR micronutrient OR micronutrients OR oligoelement OR oligoelements OR microelement OR microelements OR vitamin OR vitamins OR oil OR oils OR “trace element” OR “trace elements”	OR	safety OR hazard OR hazards OR risk OR risks OR microorganism OR microorganisms OR pathogen OR pathogens OR contaminant OR contaminants OR contamination OR contaminations OR chemical OR chemicals OR toxic OR toxics OR toxicity OR metal OR metals OR toxin OR toxins OR allergy OR allergies OR allergen OR allergens OR allergic OR allergenic OR sensitization OR sensitisation OR cross-reactivity OR anaphylactic OR anaphylaxis OR poisoning OR poison OR compound OR compounds OR pesticide OR pesticides OR residual OR residue OR residues OR antibiotic OR antibiotics OR antiparasitic OR antiparasitics OR mycotoxin OR mycotoxins OR dioxin OR dioxins OR polluting OR pollutant OR pollutants

## Data Availability

Not applicable.
